# Clinical features of *Tropheryma Whipplei* in pediatric pneumonia: an mNGS and tNGS-based case-control study

**DOI:** 10.3389/fcimb.2026.1753963

**Published:** 2026-03-17

**Authors:** Yijia Pan, Na Du, Yong Liu, Ming Wu, Sijia Hao, Yulu He, Yanfang Jiang

**Affiliations:** 1 Genetic Diagnosis Center, The First Hospital of Jilin University, Changchun, Jilin, China; 2 Department of Infectious Diseases, The First Hospital of Jilin University, Changchun, Jilin, China

**Keywords:** bronchoalveolar lavage fluid, metagenomic next-generation sequencing, pediatric pneumonia, targeted next-generation sequencing, *Tropheryma whipplei*

## Abstract

**Introduction:**

*Tropheryma whipplei* (TW), which causes Whipple disease, has recently been associated with respiratory diseases, particularly pneumonia. To understand its role in pediatric pneumonia, this study analyzed the clinical and pathogenetic characteristics of TW in pediatric pneumonia patients.

**Methods:**

We utilized metagenomic and targeted next-generation sequencing (mNGS/tNGS) data from 3,759 pediatric bronchoalveolar lavage fluid (BALF) samples (2023-2024). This case-control study included 103 TW-positive pediatric pneumonia patients (59 with severe pneumonia, SPTW+; 44 with mild pneumonia, MPTW+), along with 206 TW-negative pneumonia patients as controls (118 with severe pneumonia, SPTW-; 88 with mild pneumonia, MPTW-).

**Results:**

Through inter-group comparisons, the results showed that TW-positive patients were younger and had lower BMIs than controls, with shorter hospital stays and milder inflammation. Severe TW-positive cases showed more localized right-lung lesions, less pleural effusion, and more bronchial involvement. *Mycoplasma pneumoniae* co-detection was frequent (86.4%), along with *Moraxella catarrhalis*, *human bocavirus type 1*, and *rhinovirus A*.

**Discussion:**

TW-positive pediatric pneumonia presents with milder symptoms, suggesting that TW may act as a colonizer rather than a primary pathogen. Consequently, antimicrobial treatment specifically targeting TW may not be immediately warranted at detection. These results provide important insight for the individualized treatment of pediatric pneumonia with TW positive.

## 1 Introduction

*Tropheryma whipplei* (TW) is a Gram-positive rod-shaped bacterium belonging to the *Actinomycetes* phylum (Boumaza et al., 2022). It was first identified in 1991 through the amplification of bacterial 16S ribosomal DNA from small intestine biopsy specimens of a patient with classic Whipple’s disease (Relman et al., 1992). Currently, TW can be detected in diverse clinical specimens, such as feces, blood, saliva, bronchoalveolar lavage fluid (BALF), urine, cerebrospinal fluid, aqueous humor, synovial fluid, skin, and lymph nodes (Dolmans et al., 2017).

TW is the causative agent of Whipple’s disease, which is a chronic multisystem infection first described by George Hoyt Whipple in 1907, predominantly affecting the gastrointestinal tract (GH WJBJHH, 1907). In recent years, it has been reported that TW also causes acute infections, and manifest as gastroenteritis, pneumonia, or fever (Boumaza et al., 2022).

Recently, the potential association between TW and respiratory diseases, especially pneumonia, has garnered increasing attention (Marth et al., 2016). This bacterium was first detected in the BALF of children suffering from interstitial lung disease in 2007 (Harris et al., 2007). Subsequent researches have strengthened the evidence for its role in pneumonia. For example, Fenollar et al. provided crucial evidence by demonstrating TW as the sole bacterium in the BALF sample of a patient with severe pneumonia, establishing a direct etiological link (Fenollar et al., 2012). Furthermore, recent case reports suggest that TW can act as an independent pathogen causing acute pneumonia (Zhang and Xu, 2021). Supporting this notion, a cross-sectional study indicated that TW should be considered a potential contributor to specific pulmonary pathologies (Lin et al., 2022).

The detection of TW in pneumonia has been promoted by the emerging metagenomic next-generation sequencing (mNGS) technique, a powerful diagnostic tool for identification of a wide spectrum of pathogens (Gu et al., 2021). Its broad pathogen coverage and high timeliness have facilitated its widespread application in diagnosing clinical infections (Zhou et al., 2022). Furthermore, targeted next-generation sequencing (tNGS) employs polymerase chain reaction (PCR) or hybridization capture for specific enrichment prior to sequencing (Yin et al., 2024), making this technique more affordable and commonly used in clinical diagnosis.

Despite the enhanced detection of TW afforded by mNGS and tNGS (Lai et al., 2024), clinical research on TW-positive pneumonia, particularly in the pediatric population, remains notably scarce and warrants urgent investigation (Fang et al., 2023). Therefore, this case-control study utilized mNGS and tNGS technologies to analyze clinical data from pediatric community-acquired pneumonia (CAP) patients who underwent BALF testing at the First Hospital of Jilin University. We analyzed 3,759 BALF samples from pediatric patients tested by either mNGS or tNGS at our institution from 2023 to 2024. Among them, we enrolled 103 children aged 3-16 years with pneumonia who tested positive for TW, along with 206 pneumonia children tested negative as controls. Then, patients were stratified into four groups based on TW detection status and pneumonia severity: TW-positive severe pneumonia (SPTW+, n=59), TW-negative severe pneumonia (SPTW-, n=118), TW-positive mild pneumonia (MPTW+, n=44), and TW-negative mild pneumonia (MPTW-, n=88). By comparing inter-group differences in baseline characteristics, clinical manifestations, laboratory parameters, and imaging features among patients with the same disease severity, this study aims to analyze the correlation between TW detection status and disease severity. The findings are expected to provide a foundation for optimizing therapeutic strategies for TW-positive pediatric pneumonia.

## 2 Materials and methods

### 2.1 Research subjects screening and grouping

Between 2023 and 2024, among 3,759 BALF samples from pediatric patients tested by either mNGS or tNGS at the First Hospital of Jilin University, 130 samples (3.46%) were tested positive for TW. These positive results corresponded to 130 distinct patients. In this retrospective analysis, we excluded patients younger than 3 years of age (n=17), non-community acquired pneumonia cases (n=5), and those with missing clinical data (n=5). The exclusion of children under 3 years of age was based on established physiological and methodological considerations. The children during this age group undergo rapid development with immature immune, respiratory, and neurological systems, leading to significant differences in laboratory reference ranges compared to older children. Furthermore, obtaining high-quality lower respiratory tract specimens like BALF is technically more challenging and carries higher relative risks in very young children (<3 years), potentially affecting the consistency and reliability of pathogen detection. Therefore, to ensure cohort homogeneity, enhance the validity of diagnostic sampling, and enable accurate interpretation of laboratory data within more comparable laboratory baselines, we restricted our study population to children aged 3 years and above.

**Figure 1:**
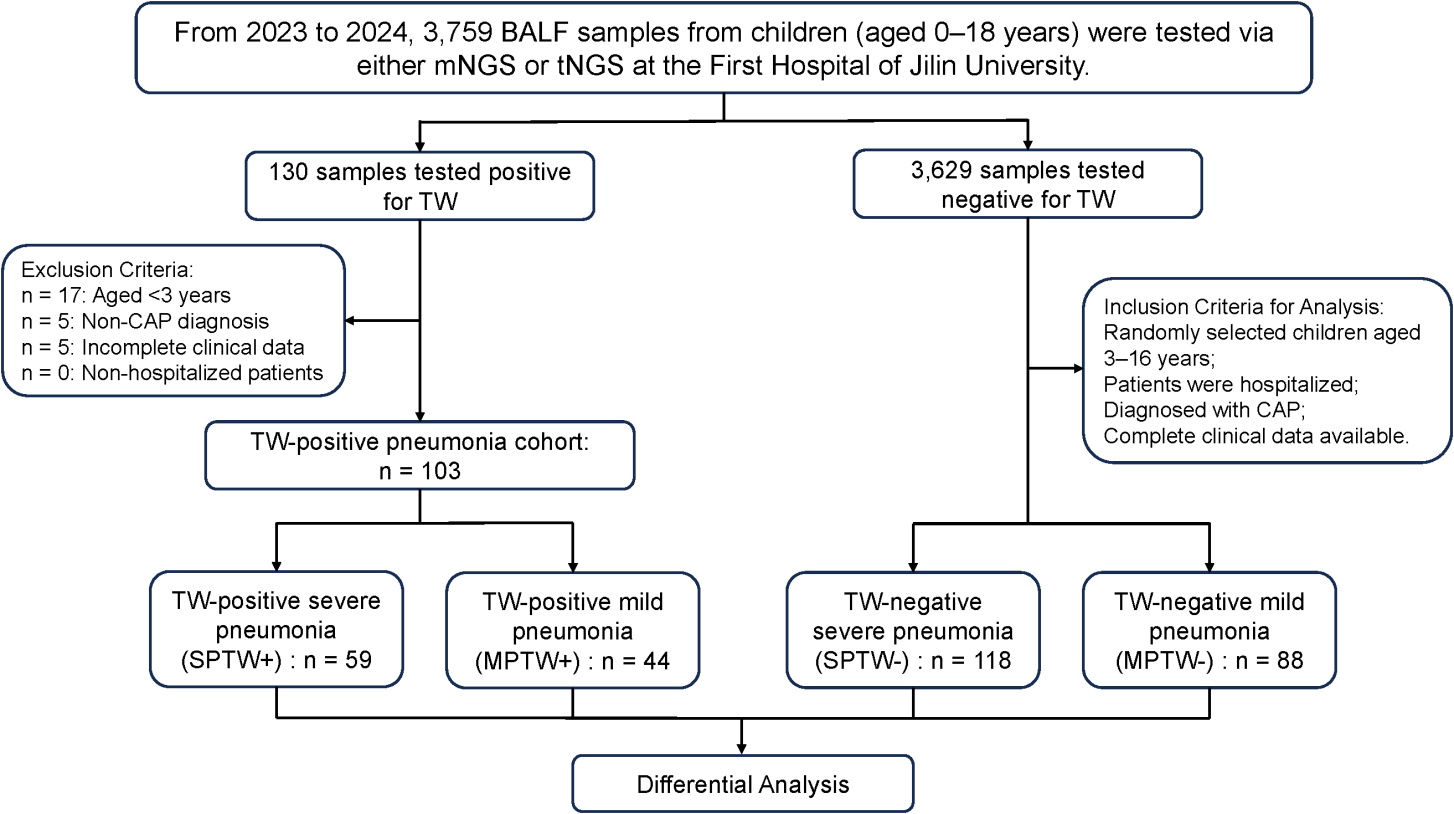
Flowchart of participants.

Consequently, we enrolled 103 hospitalized children aged 3-16 years with pneumonia who tested positive for TW. According to Guidelines for the management of community-acquired pneumonia in children (2024 revision) (Subspecialty Group of Respiratory tSoP, Chinese Medical Association, Editorial Board CJoP, Pediatrics CMEACo, 2024) (**Table 1**), these TW-positive patients were classified into two groups: 59 with severe pneumonia (SPTW+) and 44 with mild pneumonia (MPTW+). Subsequently, according to pneumonia severity, we performed stratified random sampling to select hospitalized pneumonia children aged 3-16 years who tested negative for TW by either mNGS or tNGS. These TW-negative patients served as control and were also classified into two groups: 118 with severe pneumonia (SPTW-) and 88 with mild pneumonia (MPTW-) (**Figure 1**).

**Table 1:** Severity assessment of pneumonia in children.

Assessment item	Mild pneumonia	Severe pneumonia
General condition	Good	Poor
Refusal to eat or signs of dehydration	Absent	Present
Consciousness disturbance	Absent	Present
Respiratory rate	Normal or slightly increased	Significantly increased^*^
Cyanosis	Absent	Present
Dyspnoea	Absent	Present
Oxygen saturation	Normal	≤0.92 (sea level) or ≤0.90 (high altitude)
Extent of lung infiltration	≤1/3 of a single lobe	Multiple lobes or ≥2/3 of a single lobe
Pleural effusion	Absent	Present
Extrapulmonary complications	Absent	Present

Mild pneumonia is defined by meeting all the criteria listed under the “Mild pneumonia ” column. Severe pneumonia is defined by the presence of any one of the criteria listed under the “Severe pneumonia ” column.

^*^Significantly increased respiratory rate is defined as ≥70 breaths/min for infants and ≥50 breaths/min for older children.

Inclusion criteria: (1) Aged 3–16 years; (2) Patients were hospitalized; (3) Clinical diagnosis of community-acquired pneumonia (CAP); (4) Positive for TW by mNGS or tNGS (for the TW-positive group); (5) Negative for TW by mNGS or tNGS (for the TW-negative group); (6) Availability of complete clinical data.

Exclusion criteria: (1) Aged <3 years; (2) Non-hospitalized patients; (3) Diagnosed with conditions other than CAP; (4) Incomplete clinical data.

### 2.2 mNGS and tNGS sequencing

BALF specimens were collected according to standard clinical protocols. Immediately following collection, samples underwent heat inactivation at 65°C for 30 minutes. For mechanical lysis, 0.5 mL aliquots were combined with 1g of 0.5-mm glass beads in 1.5 mL microcentrifuge tubes and subjected to vigorous agitation (2800-3200 rpm, 30 min) using a horizontal vortex platform. After centrifugation, 0.3 mL of supernatant was transferred to fresh tubes for DNA extraction with the TIANamp Micro DNA Kit (DP316, TIANGEN BIOTECH) according to manufacturer specifications. Extracted DNA concentrations were quantified using Qubit 4.0 (Thermo Fisher Scientific, MA, USA). Total RNA was extracted using QIAamp UCP pathogen minikit (Qiagen, Valencia, CA, USA) before being subjected to human rRNA depletion (Vazyme, Nanjing, China). For the creation of cDNA, 10 µl of purified RNA were employed. Metagenomic libraries were prepared with the QIAseq Ultralow Input Library Kit (QIAGEN, Hilden, Germany) (Ji et al., 2020). Inspected and qualified library was sequenced on Nextseq 550 platform (Illumina, San Diego, USA) using a 75-cycle single-end sequencing strategy.

The in-house tNGS panel was designed to detect 273 clinically significant pathogens infecting multiple systems, comprising 113 bacteria, 47 fungi, 101 viruses, and 12 parasites. Panel design leveraged multiplex PCR principles and mNGS data, incorporating pathogen prevalence information from public databases and published literature. Following nucleic acid extraction, multiple PCR was used to amplify the extracted DNA and cDNA using a pathogen-specific primer mix (Guo et al., 2021; Xie et al., 2022), with a reaction cycle as follows: pre-denaturation at 95 °C for 3 minutes, denatured at 95 °C for 20 seconds, and annealed at 60 °C for 4 minutes, a total of 25 cycles were run, with the reaction terminating at 16 °C after the cycle was stopped and extended for 4 minutes at 72 °C. Then, barcode primer was used for the second round of PCR amplification, which was conducted using the first round’s product as a template (pre-denaturation at 95°C for 3 minutes; denatured at 95 °C for 15 seconds; annealing at 58°C for 15 seconds; and extended at 72 °C for 1 minute). After 7 cycles, the cycle was extended for 10 minutes at 72 °C, and the reaction terminated at 10 °C. The concentration of the generated library was assessed using the Qubit 4.0 nucleic acid fluorometric assay and its corresponding Qubit dsDNA HS Assay kit (ThermoFisher, USA) upon purification. For 50 single-ended sequencing cycles, the library was put onto the Illumina Nextseq CN500 sequencer.

### 2.3 Bioinformatics analysis

High-quality sequencing data were generated by removing low-quality reads (Q score cutoff, 20), duplicate reads, adapter contamination, and those shorter than 40 bp using Trimmomatic, followed by computational subtraction of human host sequences mapped to the human reference genome (hg38) using Burrows-Wheeler Alignment (Li and Durbin, 2009). The remaining sequences were classified into four categories: viruses, bacteria, fungi and parasites. Classification reference databases were downloaded from National Center for Biotechnology Information, and contain 25,863 pathogens, including 12,142 bacteria, 2,680 fungi, 10,061 viruses (including DNA and RNA viruses), 654 parasites, 206 mycobacteria, and 120 mycoplasma/chlamydia.

### 2.4 Criteria for a positive mNGS/tNGS result

For each microbial taxonomy, the stringently mapped read number (SMRN) was normalized to 20 million total sequencing reads, yielding the standardized SMRN (SDSMRN). Positive mNGS/tNGS results were reported according to established criteria (Qin et al., 2021): (1) SDSMRN ≥3 for bacteria (excluding mycobacteria), fungi, DNA viruses, Mycoplasma/Chlamydia spp., and Nocardia spp.; (2) SDSMRN ≥1 for RNA viruses; (3) SDSMRN ≥100 for parasites; (4) SDSMRN ≥1 (or genus-level SDSMRNG ≥1) for Mycobacterium tuberculosis complex.

### 2.5 Statistical analysis

All statistical analyses were performed using SPSS 27.0 and R version 4.4.2. Continuous variables were presented as median (interquartile range). Categorical variables were summarized as counts and percentages. Group comparisons were conducted using the Student’s t-test or Man-Whitney U test for continuous variables, and the Fisher’s exact test for categorical variables, as appropriate. A two-tailed p-value < 0.05 was considered statistically significant.

## 3 Results

### 3.1 General characteristics of *Tropheryma whipplei*-positive children and comparison with controls

The median age of children in the SPTW+ group was 5.92 years and 6.03 years in the MPTW+ group. Sex distribution was balanced across groups. The predominant clinical manifestations included cough, fever, and expectoration. Gastrointestinal symptoms, such as abdominal pain, diarrhea and vomiting, were present in 16 patients (**Table 2**).

**Table 2:** The demographic characteristics and clinical manifestations of pneumonia patients with *Tropheryma whipplei* positive and negative in BALF.

Characteristic	Sever pneumonia (n=177)			Mild pneumonia (n=132)		
	TW (n=59)	Controls (n=118)	p value	TW (n=44)	Controls (n=88)	p value
Demographics						
Age (years)	5.92 (5.12,7.40)	7.22 (5.57,9.42)	0.001^*^	6.03 (4.75,7.73)	7.71 (6.31,10.00)	0.001^*^
Length of stay (days)	10.00 (9.00,12.50)	12.00 (9.00,15.75)	0.014^*^	7.00 (1.00,10.25)	9.00 (7.00,11.00)	0.011^*^
BMI (kg/m^2^)	14.88 (13.84,15.95)	15.56 (14.12,18.05)	0.029^*^	15.05 (14.00,16.90)	15.80 (14.76,17.87)	0.113^*^
Gender, n (%)			0.529			0.580
Male	31 (52.54)	56 (47.46)		24 (54.55)	42 (47.73)	
Female	28 (47.46)	62 (52.54)		20 (45.45)	46 (52.27)	
Deliver mode, n (%)			0.258			**0.001**
Eutocia	32 (57.14)	56 (47.46)		28 (70.00)	33 (37.93)	
Caesarean section	24 (42.86)	62 (52.24)		12 (30.00)	54 (62.07)	
Clinical manifestations						
Cough, n (%)	57 (96.61)	114 (96.61)	1.000	44 (100.00)	87 (98.86)	1.000
Expectoration, n (%)	33 (55.93)	63 (53.39)	0.873	25 (56.82)	61 (69.32)	0.178
Dyspnoea, n (%)	0 (0.00)	27 (22.88)	**<0.001**	0 (0.00)	0 (0.00)	NA
Weight loss, n (%)	1 (1.69)	0 (0.00)	0.333	2 (4.55)	3 (3.41)	1.000
Fever, n (%)	54 (91.53)	104 (88.14)	0.611	36 (81.82)	87 (98.86)	**<0.001**
Chest pain, n (%)	4 (6.78)	10 (8.47)	0.777	1 (2.27)	3 (3.41)	1.000
Abdominal pain, n (%)	4 (6.78)	11 (9.32)	0.776	2 (4.55)	7 (7.95)	0.717
Diarrhea, n (%)	1 (1.69)	5 (4.24)	0.665	2 (4.55)	5 (5.68)	1.000
Hemoptysis, n (%)	1 (1.69)	1 (0.85)	1.000	0 (0.00)	0 (0.00)	NA
Vomiting, n (%)	5 (8.47)	14 (11.86)	0.611	2 (4.55)	15 (17.05)	0.054
Neurological, n (%)	1 (1.69)	16 (13.56)	**0.013**	0 (0.00)	0 (0.00)	NA

^*^Group comparisons were conducted with Mann-Whitney U test.

Others were conducted with Fisher’s exact test.

NA, Not applicable.Bold values indicate significant P values.

Compared to the SPTW- group, children in the SPTW+ group were significantly younger (5.92 vs. 7.22 years, *P* = 0.001). A similar finding was observed when comparing the MPTW+ and MPTW- groups (6.03 vs. 7.71 years, *P* = 0.001). With either severe or mild pneumonia, TW-positive children had significantly fewer hospital stay days than TW-negative children (10.00 vs 12.00, *P* = 0.014 for severe groups; 7.00 vs. 9.00, *P* = 0.011 for mild groups). Additionally, SPTW+ children had significantly lower BMIs compared to the SPTW- group (14.88 vs. 15.56, *P* = 0.029). Children in the SPTW- group demonstrated higher incidence of dyspnea (*P* < 0.001) and neurological symptoms (*P* = 0.013), such as dizziness, headache, somnolence, listlessness, or altered consciousness, compared to the SPTW+ group. Conversely, children in the MPTW- group were significantly manifested higher incidence of fever (*P* < 0.001) than those in the MPTW+ group.

### 3.2 Laboratory characteristics

All the patients underwent relevant laboratory testing before treatment. The results are presented in **Table 3**. The main laboratory features in children with TW-positive pneumonia were elevated absolute monocyte count (MO), platelet count (PLT), erythrocyte sedimentation rate (ESR), C-reactive protein (CRP), lactate dehydrogenase (LDH), alanine aminotransferase (ALT), alongside decreased albumin (ALB) level compared to normal ranges.

**Table 3:** Laboratory examination of patients with *Tropheryma whipplei* positive and negative in BALF.

Characteristic	Sever pneumonia (n=177)			Mild pneumonia (n=132)		
	TW (n=59)	Controls (n=118)	p value	TW (n=44)	Controls (n=88)	p value
WBC (×10^9^/L)	8.13 (6.41,11.36)	10.07 (7.25,14.38)	**0.018**	7.52 (6.36, 11.70)	9.39 (6.50, 12.61)	0.307
NE (×10^9^/L)	4.81 (3.44, 6.66)	6.57 (4.24, 10.72)	**<0.001**	4.10 (2.66, 6.64)	5.30 (3.41, 8.37)	0.057
LY (×10^9^/L)	2.55 (1.93, 3.65)	1.92 (1.01, 2.85)	**<0.001**	2.96 (2.09, 3.76)	2.58 (1.67, 3.60)	0.187
MO (×10^9^/L)	0.65 (0.48, 0.94)	0.64 (0.44, 0.99)	0.831	0.66 (0.51, 0.81)	0.70 (0.47, 0.86)	0.904
RBC (×10^12^/L)	4.52 (4.31, 4.73)	4.33 (3.96, 4.59)	**0.002**	4.62 (4.34, 4.78)	4.63 (4.46, 4.82)	0.472
HGB (g/L)	125.00 (120.00, 130.50)	121.50 (110.25, 131.00)	0.004^*^	130.50 (122.75, 136.50)	129.00 (124.00, 134.75)	0.870^*^
PLT (×10^9^/L)	331.00 (258.00, 443.50)	337.50 (263.25, 417.00)	0.517	383.50 (312.50, 466.00)	381.50 (321.25, 447.25)	0.897
ESR (mm/h)	31.00 (21.00, 41.25)	36.00 (23.75, 52.75)	0.237	25.50 (19.50, 38.25)	32.00 (23.00, 50.75)	**0.039**
CRP (mg/L)	6.45 (2.54, 14.78)	11.26 (3.12, 53.49)	**0.017**	5.30 (1.29, 10.06)	4.39 (1.65, 10.36)	0.848
LDH (U/L)	311.00 (253.50, 353.00)	322.50 (260.50, 474.25)	0.094	270.00 (250.00, 321.00)	263.00 (234.50, 293.00)	0.315
TP (g/L)	66.60 (65.10, 71.80)	65.60 (60.10, 69.00)	**0.002**	68.60 (64.60, 70.30)	70.30 (65.90, 73.35)	0.199
ALB (g/L)	40.40 (37.90, 42.00)	36.00 (32.70, 39.70)	<0.001^*^	41.20 (40.00, 43.50)	40.30 (38.45, 43.15)	0.063^*^
GLB (g/L)	27.70 (25.45, 30.55)	28.20 (25.80, 31.60)	0.497^*^	26.40 (23.70, 29.00)	28.50 (25.95, 31.60)	0.011^*^
A/G	1.44 (1.29, 1.57)	1.29 (1.05, 1.45)	**<0.001**	1.62 (1.45, 1.69)	1.43 (1.29, 1.56)	**<0.001**
ALT (U/L)	13.30 (11.20, 18.70)	18.20 (11.60, 38.00)	**0.010**	12.90 (11.60, 17.70)	15.70 (12.00, 20.45)	0.144
AST (U/L)	24.40 (21.50, 33.00)	25.30 (21.10, 39.50)	0.535	24.70 (22.90, 29.40)	21.70 (19.50, 26.00)	**0.002**
BUN (μmol/L)	3.54 (3.00, 4.09)	3.65 (2.97, 4.44)	0.395	3.38 (2.75, 4.19)	3.81 (3.09, 4.55)	0.121
CRE (μmol/L)	30.10 (25.95, 35.50)	33.70 (26.00, 39.60)	0.206^*^	30.40 (27.20, 33.80)	34.40 (29.50, 38.40)	0.011^*^
UA (μmol/L)	222.00 (173.50, 272.00)	215.00 (162.00, 265.00)	0.183	229.00 (192.00, 255.00)	218.00 (177.00, 267.00)	0.621

^*^Group comparisons were conducted with Student’s t-test. Others are conducted with Mann-Whitney U test.

WBC white blood cell count, NE neutrophil count, LY lymphocyte count, MO monocyte count, RBC red blood cell count, HGB hemoglobin, PLT platelet count, ESR erythrocyte sedimentation rate, CRP C-reactive protein, LDH lactate dehydrogenase, TP total protein, ALB albumin, GLB globulin, A/G albumin-to-globulin ratio, ALT alanine aminotransferase, AST aspartate aminotransferase, BUN blood urea nitrogen, CRE creatinine, UA uric acid.Bold values indicate significant P values.

Complete blood count parameters indicated the inflammatory state of the children. Significant differences in these parameters were observed between TW-positive and TW-negative groups, particularly in the severe pneumonia patients. Compared to the SPTW- group, the SPTW+ group exhibited significantly higher absolute lymphocyte count (LY; 2.55 vs. 1.92 × 10^9^/L, *P* < 0.001), but significantly lower white blood cell count (WBC; 8.13 vs. 10.07 × 10^9^/L, *P* = 0.018) and absolute neutrophil count (NE; 4.81 vs. 6.57 × 10^9^/L, *P* < 0.001). Simultaneously, the SPTW+ group demonstrated higher red blood cell count (RBC; 4.52 vs. 4.33 × 10¹²/L, *P* = 0.002), hemoglobin (HGB; 125.00 vs. 121.50 g/L, *P* = 0.004).

Children with TW*-*positive pneumonia exhibited attenuated systemic inflammatory responses. The SPTW+ group demonstrated significantly lower C-reactive protein (CRP) levels compared to the SPTW- group (6.45 vs. 11.26 mg/L, *P* = 0.017). Similarly, the MPTW+ group showed significantly lower erythrocyte sedimentation rate (ESR) than the MPTW- group (25.50 vs. 32.00 mm/h, *P* = 0.039).

Liver function parameters revealed significant differences between the severe and mild pneumonia groups. Compared to SPTW- controls, the SPTW+ group had significantly higher albumin (ALB) (40.40 vs. 36.00 g/L, *P* < 0.001), and albumin-to-globulin ratio (A/G) (1.44 vs. 1.29, *P* < 0.001), yet significantly lower alanine aminotransferase (ALT) (13.30 vs. 18.20 U/L, *P* = 0.010). Meanwhile, the MPTW+ group displayed higher A/G ratio (1.62 vs. 1.43, *P* < 0.001) and aspartate aminotransferase (AST) (24.70 vs. 21.70 U/L, *P* = 0.002) compared to MPTW- controls, alongside lower globulin (GLB) (26.40 vs. 28.50 g/L, *P* = 0.011).

Renal function parameters showed limited correlation with TW infection status, except that creatinine (CRE) was significantly lower in the MPTW+ group versus MPTW- controls (30.40 vs. 34.40 μmol/L, *P* = 0.011).

### 3.3 Radiological manifestations

The majority of patients underwent pulmonary computed tomography (CT) prior to treatment. Chest CT manifestations included streaky or patchy opacities, pulmonary consolidation, atelectasis, pulmonary nodules, pleural effusion, mediastinal lymphadenopathy and poor aeration (**Table 4**). Among children with severe pneumonia, the SPTW- group demonstrated more extensive pulmonary involvement and a higher incidence of pleural effusion (*P* = 0.013), while the SPTW+ group exhibited higher chances of bronchial involvement (*P* = 0.049). No significant differences in CT features were observed between the two mild pneumonia groups.

**Table 4:** Radiological manifestations of patients with *Tropheryma whipplei* positive and negative in BALF.

Characteristic	Sever pneumonia (n=170)			Mild pneumonia (n=98)		
	TW (n=56), n (%)	Controls (n=114), n (%)	p value	TW (n=24), n (%)	Controls (n=74), n (%)	p value
Bronchitis	37 (66.07)	56 (49.12)	**0.049**	11 (45.83)	34 (45.95)	1.000
Pulmonary inflammation	51 (91.07)	112 (98.25)	**0.040**	21 (87.50)	67 (90.54)	0.703
Unilateral, left	6 (10.71)	16 (14.04)	0.633	8 (33.33)	34 (45.95)	0.346
Unilateral, right	24 (42.86)	24 (21.05)	**0.004**	13 (54.17)	33 (44.59)	0.484
Bilateral	21 (37.50)	72 (63.16)	**0.002**	0 (0.00)	0 (0.00)	NA
Single lobe	13 (23.21)	21 (18.42)	0.541	21 (87.50)	67 (90.54)	0.703
Multiple lobar	38 (67.86)	91 (79.82)	0.126	0 (0.00)	0 (0.00)	NA
Pulmonary consolidation	10 (17.86)	26 (22.81)	0.551	5 (20.83)	8 (10.81)	0.296
Pulmonary atelectasis	5 (8.93)	20 (17.54)	0.170	4 (16.67)	6 (8.11)	0.254
Pulmonary emphysema	0 (0.00)	1 (0.88)	1.000	0 (0.00)	1 (1.35)	1.000
Mediastinal lymphadenopathy	8 (14.29)	20 (17.54)	0.665	2 (8.33)	9 (12.16)	1.000
Pleural effusions	7 (12.50)	34 (29.82)	**0.013**	0 (0.00)	0 (0.00)	NA
Unilateral	6 (10.71)	25 (21.93)	0.055	0 (0.00)	0 (0.00)	NA
Bilateral	1 (1.79)	9 (7.89)	0.168	0 (0.00)	0 (0.00)	NA
Pulmonary nodule	4 (7.14)	5 (4.39)	0.479	1 (4.17)	8 (10.81)	0.446
Single	4 (7.14)	3 (2.63)	0.220	1 (4.17)	5 (6.76)	1.000
Multiple	0 (0.00)	2 (1.75)	1.000	0 (0.00)	3 (4.05)	1.000
Poor aeration of pulmonary tissue	6 (10.71)	12 (10.53)	1.000	0 (0.00)	2 (2.70)	1.000

P values were calculated using the Fisher’s exact test. NA: Not applicable.Bold values indicate significant P values.

### 3.4 Pathogen detection profile

We analyzed the pathogens detected in all patients (**Table 5**). Within the TW-positive group, there was no significant difference in pathogen detection rates between severe and mild pneumonia patients (all *P* > 0.05). Notably, within the TW-negative pneumonia cohort, children with severe pneumonia are more susceptible to *Candida albicans*, whereas, *Mycoplasma pneumoniae* and *Human respirovirus 3* showed associations with mild pneumonia. Overall, *Mycoplasma pneumoniae* was the most common pathogen in both severe and mild pneumonia. We also found that *Streptococcus pneumoniae*, *Haemophilus influenzae*, *Moraxella catarrhalis*, *Staphylococcus aureus* and *Human adenovirus B3* predominantly infect patients with severe pneumonia. Similar to it, mild pneumonia is primarily caused by *Streptococcus pneumoniae*, *Haemophilus influenzae*, *Staphylococcus aureus* and *Human adenovirus B3*.

**Table 5:** The proportion and comparative analysis of respiratory pathogen profiles in SPTW+ (n=59), MPTW+ (n=44), SPTW- (n=118) and MPTW- patients (n=88) based on mNGS/tNGS.

Characteristic	TW-positive pneumonia (n=103)			TW-negative pneumonia (n=206)		
	Severe (n=59), n (%)	Mild (n=44), n (%)	p value	Severe (n=118), n (%)	Mild (n=88), n (%)	p value
Streptococcus pneumoniae	9 (15.25)	8 (18.18)	0.790	13 (11.02)	7 (7.95)	0.635
Haemophilus influenzae	10 (16.95)	6 (13.64)	0.786	11 (9.32)	8 (9.09)	1.000
Moraxella catarrhalis	9 (15.25)	4 (9.09)	0.389	4 (3.39)	2 (2.27)	1.000
Staphylococcus aureus	6 (10.17)	6 (13.64)	0.758	9 (7.63)	4 (4.55)	0.405
Pseudomonas aeruginosa	2 (3.39)	1 (2.27)	1.000	1 (0.85)	2 (2.27)	0.577
Acinetobacter baumannii	0 (0.00)	2 (4.55)	0.180	6 (5.08)	3 (3.41)	0.735
Mycoplasma pneumoniae	54 (91.53)	35 (79.55)	0.090	80 (67.80)	82 (93.18)	**<0.001**
Pneumocystis jirovecii	2 (3.39)	1 (2.27)	1.000	0 (0.00)	1 (1.14)	0.427
Candida albicans	0 (0.00)	2 (4.55)	0.180	9 (7.63)	0 (0.00)	**0.011**
Human adenovirus B3	4 (6.78)	5 (11.36)	0.491	9 (7.63)	11 (12.50)	0.342
Human bocavirus 1	4 (6.78)	4 (9.09)	0.721	3 (2.54)	2 (2.27)	1.000
Rhinovirus A	3 (5.08)	1 (2.27)	0.634	0 (0.00)	0 (0.00)	NA
Rhinovirus B	2 (3.39)	1 (2.27)	1.000	1 (0.85)	1 (1.14)	1.000
Human bocavirus 3	2 (3.39)	1 (2.27)	1.000	0 (0.00)	1 (1.14)	0.427
Human respirovirus 1	2 (3.39)	1 (2.27)	1.000	1 (0.85)	1 (1.14)	1.000
Human respirovirus 3	2 (3.39)	1 (2.27)	1.000	0 (0.00)	6 (6.82)	**0.005**
Influenza A virus H3N2	1 (1.69)	1 (2.27)	1.000	1 (0.85)	0 (0.00)	1.000
Influenza B virus	2 (3.39)	0 (0.00)	0.506	1 (0.85)	1 (1.14)	1.000

P values were calculated using the Fisher’s exact test. NA, Not applicable.Bold values indicate significant P values.

**Figure 2:**
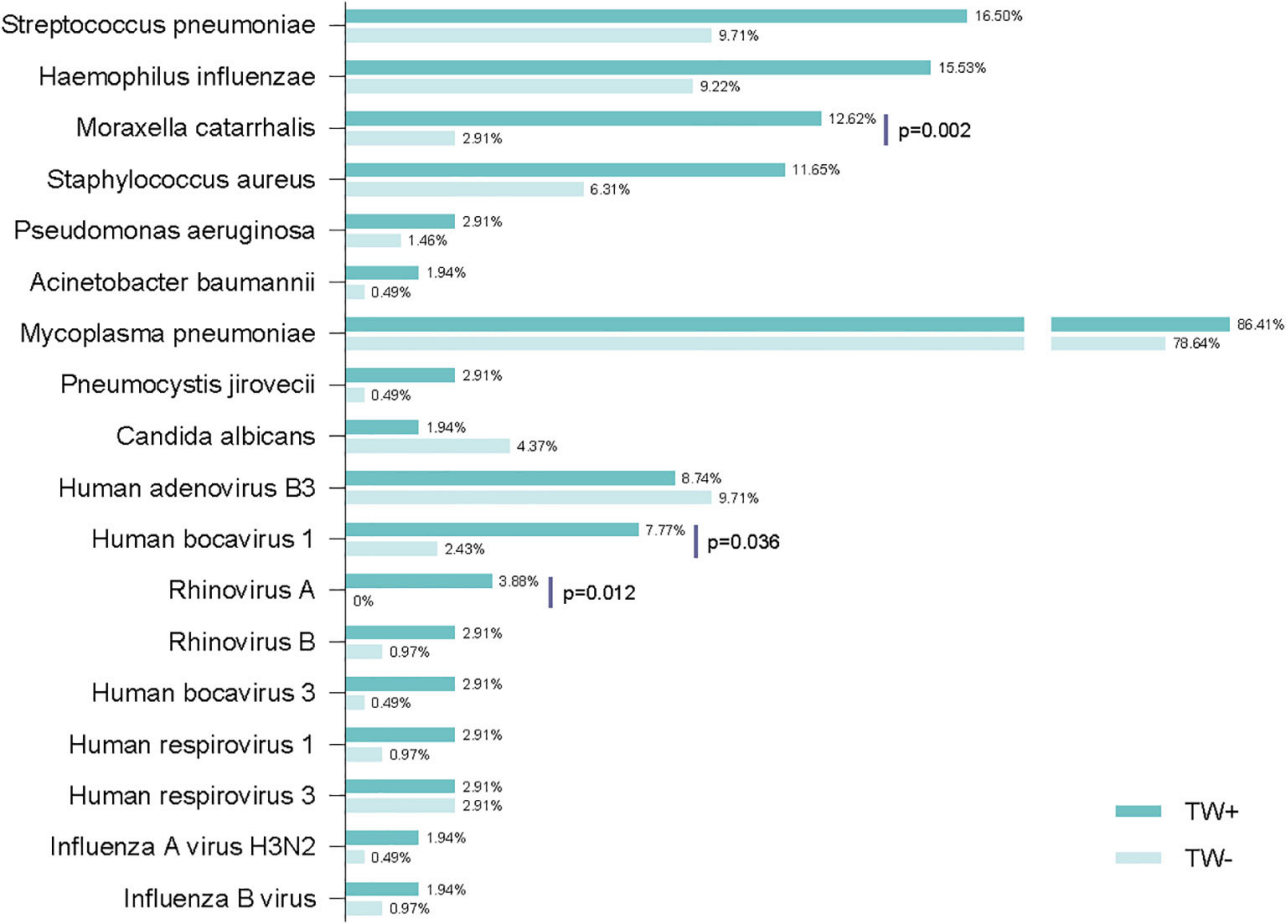
The proportion and comparative analysis of respiratory pathogen profiles in TW-positive patients (n=103) and TW-negative patients (n=206) based on mNGS/tNGS. P values were calculated using the Fisher’s exact test.

Then, we analyzed pathogens detected together with TW (**Figure 2**). Among children with TW-positive pneumonia, nearly all (86.41%) were co-detected with *Mycoplasma pneumoniae*. The predominant bacterial pathogens were S*treptococcus pneumoniae* (16.50%) and *Haemophilus influenzae* (15.53%), followed by *Moraxella catarrhalis* (12.62%) and *Staphylococcus aureus* (11.65%). The detection rates of fungi were low, with only three patients tested positive for *Pneumocystis jirovecii* (2.91%) and two for *Candida albicans* (1.94%). *Human adenovirus type 3* (8.74%) was the most commonly identified virus, succeeded by *Human bocavirus type 1* (7.77%). Critically, TW-positive children demonstrated significantly higher co-detection rates with *M. catarrhalis*, *Human bocavirus type 1*, and *Rhinovirus A* than TW-negative controls.

### 3.5 Treatment and prognosis

The treatment regimens for TW-positive and TW-negative patients within the same severity stratum (severe or mild) were consistent in our cohort, with no specific anti-TW therapy. The therapeutic regimens for pediatric patients comprised anti-infective therapy, and anti-inflammatory treatments, along with symptomatic supportive care, which were standardized based on the Guidelines for the management of community-acquired pneumonia in children (2024 revision) (Subspecialty Group of Respiratory tSoP, Chinese Medical Association, Editorial Board CJoP, Pediatrics CMEACo, 2024). For drug-resistant *M. pneumoniae* infections, azithromycin or doxycycline was administered. Third-generation cephalosporins, selected based on antimicrobial susceptibility testing, were used for mixed bacterial infections, while interferon or oseltamivir was employed for viral pneumonia. Methylprednisolone sodium succinate was routinely administered for anti-inflammatory purposes, with dosage adjusted according to disease severity. Symptomatic supportive therapy encompassed nebulized budesonide combined with terbutaline for bronchodilation, intravenous bromhexine combined with nebulized acetylcysteine for cough relief and sputum expectoration, low-molecular-weight heparin (LMWH) for anticoagulation, and nutritional support. Severe cases additionally underwent bronchoscopic intervention including repeated bronchial lavage and mucus plug removal.

Children with mild pneumonia had an approximately 7-9-day treatment course with favorable prognosis and largely resolved symptoms at discharge. Those with severe pneumonia required approximately 10-12 days of treatment, and most exhibited significant symptomatic improvement at discharge (e.g., resolution of fever, reduced cough), though some retained residual radiographic abnormalities such as prominent lung markings or minimal pleural effusion, necessitating outpatient follow-up.

## 4 Discussion

This study employed mNGS and tNGS to elucidate the clinical characteristics of TW-positive pediatric pneumonia patients. The results revealed significant differences in demographic characteristics, inflammatory indicators, imaging findings, and patterns of co-detection between TW-positive and TW-negative pediatric pneumonia patients, suggesting that TW may play a unique ecological or pathophysiological role in the pediatric airway.

TW-positive pediatric pneumonia patients demonstrated significantly younger ages and lower BMIs. These characteristics suggest a potential link to age-dependent susceptibility or malnutrition-associated immune deficiency, potentially contributing to TW colonization or infection (Makka et al., 2022). Respiratory symptoms and fever were the predominant clinical manifestations of patients, while gastrointestinal symptoms were rare, which is consistent with previous reports of TW-associated pneumonia (Guo et al., 2021). Notably, TW-positive patients with comparable pneumonia severity exhibited shorter hospital stays and milder clinical symptoms, such as fever, coughing, vomiting, dyspnea, and neurological symptoms. Similar phenomena were observed in studies of children with TW-related gastroenteritis (Raoult et al., 2010). This suggests that TW may not be an independent pathogen, and its presence may interact with the host immune microenvironment to modulate the intensity of the inflammatory response (Xiao et al., 2025).

Through comparing TW-positive and TW-negative pediatric pneumonia patients with similar clinical conditions, we found that TW-positive cases had weaker inflammatory responses, which is consistent with the aforementioned shorter disease duration. This phenomenon may be caused by the M2-polarized macrophages prompting Th2 responses and exerting anti-inflammatory effects (Luo et al., 2024), which were reported presenting in the duodenal mucosa of patients with classic Whipple’s disease (Desnues et al., 2005). Meanwhile, impaired antigen-presenting function in macrophages and dendritic cells further compromises T-cell responses (Moos et al., 2010). However, given the lack of research on the pathogenesis of TW-induced pulmonary infection, whether TW leads to impaired function in pulmonary macrophages requires further investigation.

It should be noted that among children with severe pneumonia, TW-positive patients were significantly more likely to exhibit right-sided unilateral lung involvement (42.86% vs. 21.05%, *P* = 0.004) and had significantly lower rates of pleural effusion (12.50% vs. 29.82%, *P* = 0.013) compared to the control group. This suggests a pattern of localized disease progression. This may be attributable to the fact that TW, as an obligate intracellular pathogen, resides and replicates primarily within macrophages, as opposed to undergoing rapid extracellular dissemination. Consequently, it is less likely to trigger an acute, widespread inflammatory response (Boumaza et al., 2022). Furthermore, children in the SPTW+ group demonstrated significantly higher involvement of the bronchi. This may be due to TW’s macrophage tropism, presumably facilitating its preferential colonization within Bronchus-Associated Macrophages (BAMs) (Tang et al., 2022).

TW can lead to pulmonary infections either alone or in combination with other pathogens (Tao et al., 2024). *Klebsiella pneumoniae*, *Acinetobacter baumannii*, *Pneumocystis jirovecii*, *Candida albicans* and the *Mycobacterium tuberculosis* complex are commonly co-detected pathogens in adult cases (Lai et al., 2024). However, within this pediatric pneumonia cohort, *Mycoplasma pneumoniae* exhibited the highest co-detection rate (86.41%), followed by *Streptococcus pneumoniae* (16.50%). This likely reflects that *M. pneumoniae* is a primary pathogen in pediatric pneumonia in China (Zhang et al., 2022), and *S. pneumoniae* represents the most significant bacterial pathogen for pneumonia in children beyond the neonatal period across all age groups (Subspecialty Group of Respiratory tSoP, Chinese Medical Association, Editorial Board CJoP, Pediatrics CMEACo, 2024). We postulate that infection with M. pneumoniae or S. pneumoniae in children may induce macrophage functional impairment, thereby potentially facilitating TW colonization and subsequent infection. Moreover, building on the hypothesis which we mentioned above that TW may reduce local inflammation by inducing macrophage polarization toward an M2 phenotype, it is compelling to consider the broader ecological consequences of this immunomodulation. M2-type macrophages can dampen the immune response by secreting anti-inflammatory factors IL-10, TGF-β, and interleukin-1 receptor antagonist (IL-1RA) (Boutilier and Elsawa, 2021). This functional shift not only suppresses pro-inflammatory signaling but may also reduce the phagocytic efficiency and antigen-presenting capacity of macrophages, thereby weakening the overall defensive barrier of the respiratory mucosa. Consequently, this altered immune landscape could plausibly create a permissive microenvironment in the lungs or respiratory tract, thereby facilitating co-infection with other pathogens. In short, the presence of TW may precondition the host’s immune state, creating an opportunity for subsequent invasion of other pathogens. Nevertheless, the interactions and causal relationships between TW and other pulmonary pathogens require elucidation through further mechanistic studies.

Current therapeutic agents for TW include ceftriaxone, meropenem, trimethoprim-sulfamethoxazole (TMP-SMZ), doxycycline, and hydroxychloroquine (Marth et al., 2016). Despite the absence of targeted anti-TW therapy in this study, the pediatric patients still achieved favorable outcomes. This suggests that TW may have acted as a non-dominant pathogen within the co-detected microbial milieu. However, for patients co-detected with macrolide-resistant *Mycoplasma pneumoniae*, optimization of the therapeutic regimen is warranted, such as switching from azithromycin to doxycycline. The milder clinical presentation and shorter disease course observed in TW-positive children suggest that initiating immediate targeted anti-TW therapy may not be necessary. However, given that the potential for synergistic pathogenicity between TW and other pathogens remains unelucidated, whether targeted anti-TW treatment is required in the context of co-detection requires further investigation.

As a single-center retrospective study, it is susceptible to selection bias, thus limiting the ability to establish causal relationships. Additionally, the absence of histopathological examination precluded definitive diagnosis of TW infection. Nevertheless, we employed mNGS and tNGS, implementing stringent positive and negative controls alongside rigorous quality control measures throughout the sequencing and bioinformatics analysis pipeline, ensuring the reliability of the results. Although the pathogenic mechanisms by which TW causes pneumonia remain incompletely elucidated, this clinical study provides forceful evidence supporting its role in pneumonia.

## Data Availability

The datasets presented in this study can be found in online repositories. The names of the repository/repositories and accession number(s) can be found below: https://ngdc.cncb.ac.cn/gsa, CRA033981.
